# Supplementary material for: Novel 1,4-Dihydropyridine Derivatives as Mineralocorticoid Receptor Antagonists
Source: Int J Mol Sci. 2023 Jan 26;24(3):2439. doi: 10.3390/ijms24032439 (PMC9917360; doi:10.3390/ijms24032439)
Supplement: Supplementary file 1 [file ijms-24-02439-s001.zip › ijms-2123476-supplementary.pdf]

Novel 1,4-Dihydropyridine Derivatives as Mineralocorticoid Receptor Antagonists

Felipe Luis Pérez-Gordillo [1], Natalia Serrano-Morillas [2], Luz Marina Acosta-García [2], María Teresa Aranda [1], Daniela Passeri [3], Roberto Pellicciari [3], María Jesús Pérez de Vega [1], Rosario González-Muñiz [1], Diego Alvarez de la Rosa [2,*] and Mercedes Martín-Martínez [1,*]

1 Instituto de Química Médica (IQM-CSIC), Juan de la Cierva, 3, 28006 Madrid, Spain
2 Departamento de Ciencias Médicas Básicas and Instituto de Tecnologías Biomédicas, Universidad de La Laguna, 38200 La Laguna, Tenerife, Spain
3 Tes Pharma, via P. Togliatti 20, 06073 Taverne di Corciano, Italy
* Correspondence: dalrosa@ull.edu.es (D.A.d.l.R.); mmartin@iqm.csic.es (M.M.-M.)

Supporting information

S1. Focused libraries

Table S1. 1st focused library, based on proteinogenic amino acids as element of structural diversity. L and D amino acids have been studied.

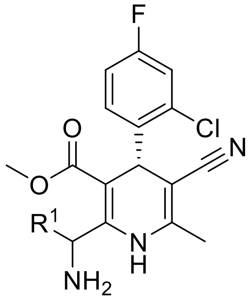

DHP	Conf.	Amino acid	R[1]
**L1.1**		Gly	H
**L1.2**	L	Ala	CH_3
**L1.3**	D		
**L1.4**	L	Val	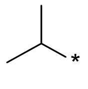
**L1.5**	D		
**L1.6**	L	Phe	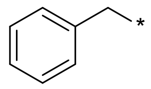
**L1.7**	D		
**L1.8**	L	Tyr	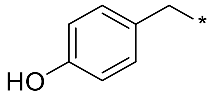
**L1.9**	D		

DHP	Config.	Amino acid	R[1]
**L1.10**	L	Asn	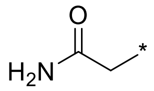
**L1.11**	D		
**L1.12**	L	Gln	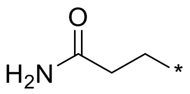
**L1.13**	D		
**L1.14**	L	Asp	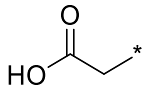
**L1.15**	D		
**L1.16**	L	Glu	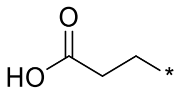
**L1.17**	D		
**L1.18**	L	Lys	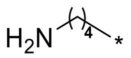
**L1.19**	D		

Table S2. 2nd focused library, starting from DHP L1.1 (Table S1) and attaching different substituents at the NH_2 at position 2 of the DHP ring.

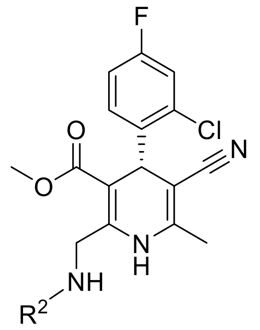

DHP	R^2	DHP	R^2
**L2.1**	$COCH_3$	**L2.5**	$COCH_2COOH$
**L2.2**	$COCH_2OH$	**L2.6**	$CO(CH_2)_2COOH$
**L2.3**	$CO(CH_2)_2OH$	**L2.7**	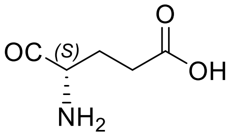
**L2.4**	COCOOH		

S2. Synthetic procedures

General: All reagents and solvents were of commercial quality. The 3-aminocrotonates and the aldehydes are commercially available. The β-keto esters are either commercially available or their preparation has been described within publications [1-6].

Reaction monitoring: TLC silica gel plates (Merck 60 F254, Spain) and a HPLC-MS Waters system composed of a 2695 separations module, a 2996 Photodiode Array (UV, 230-700 nm), a MicroMass ZQ 2000 (electrospray positive mode, m/z: 100-1500) and a Sunfire C18 Column, 100 Å, 2.5 µm, 2.1 x 50 mm; mobile phase (A:B:C), CH_3CN(A):H_2O(B):H_2O(1% TFA)(C); flux, 0.2 mL/min. Chromatographic separations: flash column, silica gel Merck 60 (230–400), HPLC-MS Waters semipreparative purification system composed of a 2767 sample manager, a SFO system fluidic organizer, a 2545 binary gradient module, a 515 auxiliary pump, a 2998 photodiode array, a 3100 Mass Detector and a Sunfire C18 Column, 100 Å, 5 µm, 19 x 150 mm; mobile phase (A:B), CH_3CN (0.1% formic acid)(A): H_2O(0.1% formic acid)(B); flux, 17 mL/min. ^1H NMR spectra: Varian INOVA-300 (300 MHz), Bruker 300 (300 MHz), Varian INOVA-400 (400 MHz) and Varian System-500 (500 MHz), with TMS as internal standard. ^{13}C NMR spectra: Varian INOVA-300 (75 MHz), Bruker 300 (75 MHz), Varian INOVA-400 (101

MHz) and Varian System-500 (126 MHz). Chemical shifts are expressed in ppm, the coupling constants are expressed in Hz. Mass spectra; Waters MicroMass ZQ 2000 (electrospray positive mode, m/z: 100-1500). Exact mass: high resolution mass spectra (ESI-HRMS) were recorded on an Agilent 6520 Q-TOF instrument.

S2.1. Synthesis of dihydropiridines

Following the methods included in the manuscript section 4.2.1.

Methyl 2-[1(S)-(benzyloxycarbonyl)amino]ethyl-4-(2-chloro-4-fluoro)phenyl-5-cyano-6-methyl-1,4-dihydropyridine-3-carboxylate (12ab) From methyl (4S)-[(benzyloxycarbonyl)amino]-3-oxopentanoate, 2-chloro-4-fluorobenzaldehyde and 3-aminocrotonate. Method A: 5 min (40 ºC), 15 min (100 ºC) and 3 days ($CHCl_3$). Yield 55%. Method B. Yield 80%. Syrup. Eluent: Hexane:EtOAc (3:2), HPLC-MS: Gradient from 30 to 95% ACN/H_2O (0.05% TFA) in 10 min, t_R=8.10 min (m/z: 484.08 $M+H^+$), this purification do not allow the isolation of diasteroisomers. HRMS (ESI pos) m/z Calculated $C_{25}H_{23}ClFN_3O_4$ 483.13611, found 483.13704 (1.92 ppm). Diasteroisomers were subsequently resolved by semi-preparative HPLC-MS. Gradient from 40 to 70% ACN/H_2O (0.1% formic acid) in 70 min (diasteroisoners A:B 1:1), FP531a (t_R=48min, m/z: 484.04 $M+H^+$) and FP531b (t_R=52min, m/z: 484.04 $M+H^+$). **12a: ^{1}H NMR** (400 MHz, $CDCl_3$, 50 ºC): δ 7.34 (m, 6H, 1-H, Ph Cbz), 7.17 (dd, *J* = 8.7, 6.1 Hz, 1H, Ph 6-H), 7.08 (dd, *J* = 8.6, 2.6 Hz, 1H, Ph 3-H), 6.91 (ddd, J = 8.7, 8.2, 2.6 Hz, 1H, Ph 5-H), 5.47 (d, *J*=6.7 Hz, 1H, NHCO), 5.44 (s, 1H, Et CH), 5.20 (s, 1H, 4-H), 5.17 (d, *J* = 12.2 Hz, 1H, Cbz CH_2), 5.09 (d, *J* = 12.2 Hz, 1H, Cbz CH_2), 3.54 (s, 3H, OCH_3), 1.96 (s, 3H, 6-CH_3), 1.55 (d, *J* = 6.7 Hz, 3H, Et CH_3). **^{13}C NMR** (101 MHz, $CDCl_3$, 50 º C): δ 166.8 (3-CO), 161.5 (d, *J* = 249.5 Hz, 4-C Ph), 157.1 (Cbz CO), 150.0 (2-C), 145.3 (6-C), 139.2 (d, *J* = 3.6 Hz, Ph 1-C), 136.1 (Cbz Ph C), 133.5 (d, *J* = 10.3 Hz, Ph 2-C), 131.6 (d, *J* = 8.8 Hz, Ph 6-C), 128.8, 128.7, 128.3(Cbz Ph CH), 118.8 (CN), 117.1 (d, *J* = 24.5 Hz, Ph 3-C), 114.8 (d, *J* = 21.2 Hz, Ph 5-C), 100.0 (3-C), 86.2 (5-C), 67.7 (Cbz CH_2), 51.4 (OCH_3), 47.4 (Et CH), 38.4 (4-C), 18.9 (Et CH_3), 18.4 (6-CH_3). **12b: ^{1}H NMR** (400 MHz, $CDCl_3$, 50 ºC): δ 7.35 (m, 5H, 6-H, Cbz), 7.21 (bs, 1H, Ph 6-H), 7.07 (dd, *J* = 8.6, 2.6 Hz, 1H, Ph 3-H), 6.87 (bs, 1H, Ph 5-H), 5.50 (bs, 1H, Et CH), 5.28 (d, *J* = 5.1 Hz, 1H, Cbz NH), 5.18 (s, 1H, 4-H), 5.16 (s, 2H, Cbz CH_2), 3.55 (s, 3H, OCH_3), 2.04 (s, 3H, 6-CH_3), 1.49 (d, *J* = 7.0 Hz, 3H, Et CH_3). **^{13}C NMR** (126 MHz, $CDCl_3$, 50 °C): δ 166.7 (3-CO), 161.4 (d, *J* = 249.2 Hz, Ph 4-C), 156.8 (Cbz CO), 150.3 (2-C), 145.1 (6-C), 139.6 (d, *J* = 3.5 Hz, Ph 1-C), 136.0 (Cbz Ph C), 132.9 (d, *J* = 10.4 Hz, Ph 2-C), 131.6 (d, *J* = 8.8 Hz, Ph 6-C), 128.8, 128.7, 128.2 (Cbz Ph CH), 118.9 (CN), 116.7 (d, *J* = 24.3 Hz, Ph 3-C), 115.0 (d, *J* = 21.1 Hz, Ph 5-C), 100.0 (3-C), 86.7 (5-C), 67.7 (Cbz CH_2), 51.5 (OCH_3), 47.7 (Et CH), 37.5 (4-C), 19.0 (Et CH_3), 18.5 (6-CH_3).

Methyl 2-[1(S)-(benzyloxycarbonyl)amino]isobutyl-4-(2-chloro-4-fluoro)phenyl-5-cyano-6-methyl-1,4-dihydropyridine-3-carboxylate (13ab). From (4S)-methyl-4-[(benzyloxycarbonyl)amino]-5-methyl-3-oxohexanoate, 2-chloro-4-fluorobenzaldehyde and 3-aminocrotonate. Method A: 3 min (40 ºC), 3 min (100 ºC) and 2 days ($CHCl_3$). Yield

59%. Syrup. Method B: Yield 74%. Syrup. Eluent: Hexane:EtOAc (3:1). It is obtained as a diateroisomeric mixture that could not be resolved. HPLC-MS: Gradient from 30 to 95% ACN/H_2O (0.05% TFA) in 10 min, t_R: 9.06, (m/z: 512.06 $M+H^+$). HRMS (ESI pos) m/z Calculated $C_{27}H_{27}ClFN_3O_4$ 511.16741, found 511.16597 (-2.83 ppm). **^{1}H NMR** (500 MHz, $CDCl_3$) δ 7.73 (bs, 2H, 1-H) 7.39 – 7.29 (m, 10H, Cbz), 7.22 (dd, *J* = 8.7, 6.0 Hz, 2H, Ph 6-H), 7.09-7.05 (m, 2H, Ph 3-H), 6.93-6.87 (m, 2H, Ph 5-H), 5.88 (bs, 1H, NHCO), 5.69 (bs, 1H, NHCO), 5.24 (s, 1H, 4-H), 5.21 (s, 1H, 4-H), 5.19-5.06 (m, 6H, Cbz CH_2, ^{i}Bu 1-H), 3.54 (s, 3H, OCH_3), 3.52 (s, 3H, OCH_3), 2.02 (s, 3H, 6-CH_3), 1.97 (s, 3H, 6-CH_3), 1.04-0.97 (m, 14H, ^{i}Bu 2-H, CH_3). **^{13}C NMR** (126 MHz, $CDCl_3$): δ 167.2 (3-CO), 161.4 (d, *J* = 249.5 Hz, Ph 4-C), 161.4 (d, *J* = 249.1 Hz, Ph 4-C), 157.7, 157.6 (Cbz CO), 148.8 (2-C), 145.5, 145.27 (6-C), 139.7 (d, *J* = 3.6 Hz, Ph 1-C), 139.5 (d, *J* = 3.7 Hz, Ph 1-C), 136.2, 136.1 (Cbz Ph C), 133.2 (d, *J* = 10.3 Hz, Ph 2-C), 133.1 (d, *J* = 10.1 Hz, Ph 2-C), 131.6 (d, *J* = 8.8 Hz, Ph 6-C), 131.5 (d, *J* = 8.9 Hz, Ph 6-C), 128.8, 128.7, 128.60, 128.59, 128.1 (Cbz Ph CH), 119.0, 118.9 (CN), 116.9 (d, *J* = 24.1 Hz, Ph 3-C), 116.8 (d, *J* = 24.6 Hz, Ph 3-C), 114.9 (d, *J* = 21.0 Hz, Ph 5-C), 101.7, 101.6 (3-C), 86.3, 86.1 (5-C), 67.6, 67.5 (Cbz CH_2), 58.1, 57.6 (^{i}Bu 1-C) 51.5, 51.4 (OCH_3), 38.0, 37.7 (4-C), 29.8, 29.5 (^{i}Bu 2-C), 26.0, 24.9, 20.0, 19.8, 19.6, 19.2 (^{i}Bu CH_3), 18.4, 18.3 (6-CH_3).

***tert*-Butyl-2-[(benzyloxycarbonyl)amino]methyl-4-(2-chloro-4-fluoro)phenyl-5-cyano-6-methyl-1,4-dihydropyridine-3-carboxylate (15).** From tert-butyl 4-[(benzyloxycarbonyl)amino]-3-oxo-butanoate, 2-chloro-4-fluorobenzaldehydey and 3-aminocrotonate. Method A: 5 min (40 ºC), 10 min (100 ºC) and 2 days ($CHCl_3$). Yield 14%. Syrup. Purified by isocratic semi-preparative HPLC-MS, 55% CH_3CN (0.1% formic acid): 45% H_2O (0.1% formic acid) (t_R=39 min). HPLC-MS: Gradient from 30 to 95% ACN/H_2O (0.05% TFA) in 10 min, t_R: 9.80, (m/z: 512.06 $M+H^+$). HRMS (ESI pos) m/z Calculated $C_{27}H_{27}ClFN_3O_4$ 511.16741, found 511.16622 (-2.33 ppm). **^{1}H RMN** (500 MHz, CDCl3): δ 7.45 – 7.32 (m, 5H, Cbz), 7.20-7.17 (m, 2H, 1-H, Ph 6-H), 7.10 (ddd, *J* = 8.6, 2.6, 0.7 Hz, 1H, Ph 3-H), 6.93 (ddd, *J* = 8.6, 8.3, 2.6 Hz, 1H, Ph 5-H), 5.75 (t, *J* = 6.5 Hz, 1H, NHCO), 5.15 (s, 2H, Cbz CH_2), 5.12 (s, 1H, 4-C), 4.29 (m, 2H, 2-CH_2), 2.01 (s, 3H, 6-CH_3), 1.23 (s, 9H, ^{t}Bu CH_3). **^{13}C RMN** (126 MHz, CDCl3): δ 166.1 (3-CO), 161.3 (d, *J* = 249.4 Hz, Ph 4-C), 158.6 (Cbz CO), 145.5, 145.4 (2-C, 6-C), 139.3 (Ph 1-C), 136.1 (Cbz Ph C), 133.2 (d, *J* = 10.3 Hz, Ph 2-C), 131.4 (d, *J* = 8.8 Hz, Ph 6-C), 128.8, 128.6, 128.2 (Cbz Ph CH), 119.1 (CN), 116.9 (d, *J* = 24.5 Hz, Ph 3-C), 114.7 (d, *J* = 21.1 Hz, Ph 5-C), 103.6 (3-C), 85.4 (5-C), 81.4 (^{t}Bu C), 67.7 (Cbz CH_2), 41.2 (2-CH_3), 37.8 (4-C), 28.1 (^{t}Bu CH_3), 18.5 (6-CH_3).

Methyl 2-[(benzyloxycarbonyl)amino]methyl-5-carbamoil-4-(2-chloro-4-fluoro)phenyl-6-methyl-1,4-dihydropyridine-3-carboxylate (17). From methyl-4-[(benzyloxycarbonyl)amino]-3-oxo-butanoate, 2-chloro-4-fluorobenzaldehyde and 3-amino-2-butenamida. Method A: 5 min (40 ºC), 10 min (100 ºC) and 2 days ($CHCl_3$). Yield 29%. Syrup. Eluent: Hexane:EtOAc (1:9). HPLC-MS: Gradient from 30 to 95% ACN/H_2O (0.05% TFA) in 10 min, t_R = 5.86, (m/z: 488.06 $M+H^+$). HRMS (ESI pos) m/z Calculated $C_{24}H_{23}ClFN_3O_5$ 487.13103, found 487.13160 (1.18 ppm). **^{1}H NMR** (500 MHz, $CDCl_3$): δ 7.40 – 7.31 (m, 6H, Cbz Ph and Ph 6-H), 7.08 (s, 1H, 1-H), 6.99 (dd, *J* = 8.6, 2.6 Hz, 1H, Ph 3-H), 6.89 (ddd, *J* = 8.7, 8.3, 2.6 Hz, 1H, Ph 5-H), 5.74 (t, *J* = 6.7 Hz, 1H, NHCO), 5.51 (bs,

2H, 5-$CONH_2$), 5.13 (s, 1H, 4-H), 5.13 (s, 2H, Cbz CH_2), 4.37 (dd, *J* = 14.9, 6.4 Hz, 1H, 2-CH_2), 4.23 (dd, *J* = 15.0, 6.9 Hz, 1H, 2-CH_2), 3.61 (s, 3H, 3-$COOCH_3$), 2.24 (s, 3H, 6-CH_3). ^{13}**C NMR** (126 MHz, $CDCl_3$): δ 169.9 (5-CO), 167.6 (3-CO), 161.2 (d, *J* = 249.6 Hz, Ph 1-C), 158.3 (Cbz CO), 146.9 (3-C), 141.1 (d, J = 2.7 Hz, Ph 1-C), 140.9 (5-C), 136.1 (Cbz Ph C), 132.3 (d, *J* = 8.7 Hz, Ph 6-C), 131.3 (d, *J* = 10.1 Hz, Ph 2-C), 128.8, 128.5, 128.2 (Cbz Ph CH), 116.3 (d, *J* = 24.7 Hz, Ph 3-C), 115.6 (d, *J* = 21.0 Hz, Ph 5-C), 106.2 (6-C), 102.4 (2-C), 67.5 (Cbz CH_2), 51.3 (3-$COOCH_3$), 41.4 (2-CH_2), 37.2 (4-C), 19.2 (6-CH_3).

S2.2. Catalytic hydrogenation

General method: To a solution of the benzyloxicarbonyl derivative (0.1 mmol) in MeOH (10 mL) at 0°C is added Pd/C (10%). The suspension is hydrogenated at room temperature and 25 psi during 2h 30 min. After filtration, the solvent is evaporated to dryness.

Methyl 2-aminomethyl-4-(2-chloro-4-fluoro)phenyl-5-cyano-6-methyl-1,4-dihydropyridine-3- carboxylate (18). From **11**. Yield: 93%. Amorphous solid. HPLC-MS: Gradient from 15 to 95% ACN/H_2O (0.05% TFA) in 10 min, t_R = 4.40, (m/z: 336.35 $M+H^+$). This compound decomposed over time.

Methyl 2-[(S)-2-Amino-5-*terc*-butoxi-5-oxopentanamido]methyl-5-cyano-4-(2-chloro-4-fluoro)phenyl-6-methyl-1,4-dihydropyridine-3-carboxylate (23ab). From **22ab**. Eluent: DCM:MeOH (20:1). Yield 93% (diasteroisomers A:B 1:1) . Syrup. Diasteroisomer A: $[\alpha]_D^{20}$= -86.8° (*c* 0.90, $CHCl_3$). HPLC-MS: Gradient from 15 to 95% ACN/H_2O (0.05% TFA) in 15 min, t_R: 5.17 (m/z: 521.69 $M+H^+$). HRMS (ESI pos) m/z Calculated $C_{25}H_{30}ClFN_4O_5$ 520.18888, found 520.19028 (2.7 ppm). **23a:** ^{1}**H NMR** (400 MHz, $CDCl_3$): δ 8.14 (t, *J* = 6.6 Hz, 1H, CONH), 7.98 (s, 1H, 1-H), 7.21 (dd, *J* = 8.7, 6.1 Hz, 1H, Ph 6-H), 7.07 (dd, *J* = 8.6, 2.6 Hz, 1H, Ph 3-H), 6.95 (ddd, *J* = 8.7, 7.8, 2.6 Hz, 1H, Ph 5-H), 5.15 (s, 1H, 4-H), 4.55 (dd, *J* = 14.3, 6.6 Hz, 1H, 2-CH_2), 4.43 (dd, *J* = 14.3, 6.6 Hz, 1H, 2-CH_2), 3.56(s, 3H, OCH_3), 3.49 (dd, *J* = 7.6, 4.9 Hz, 1H, Pen 2-H, 2.37 (m, 1H, Pen 4-H), 2.34 (m, 1H, Pen 4-H), 2.12 (dtd, *J* = 14.9, 7.5, 4.9 Hz, 1H, Pen 3-H), 2.05 (s, 3H, 6-CH_3), 1.92 (br s, 2, Pen 2-NH_2), 1.84 (dt, *J* = 21.9, 7.6 Hz, 1H, Pen 3-H), 1.45 (s, 9H, ^{t}Bu CH_3). ^{13}**C NMR** (101 MHz, $CDCl_3$): δ 177.4, 172.6, 167.2 (CO), 161.4 (d, *J* = 249.3 Hz, Ph 4-C), 146.6, 145.7 (2-C, 6-C), 139.3 (d, *J* = 3.6 Hz, Ph 1-C), 133.0 (d, *J* = 10.4 Hz, Ph 2-C), 131.6 (d, *J* = 8.8 Hz, Ph 6-C), 119.1 (CN), 116.9 (d, *J* = 24.4 Hz, Ph 3-C), 114.9 (d, *J* = 21.0 Hz, Ph 5-C), 101.6 (3-C), 85.7 (5-C), 81.1 (^{t}Bu C), 54.5 (Pen 2-C), 51.6 (OCH_3), 39.1 (2-CH_2), 37.5 (4-C), 31.9 (Pen 4-C), 30.1 (Pen 3-C), 28.2 (^{t}Bu CH_3), 18.5 (6-CH_3). **23b**: $[\alpha]_D^{20}$= +89.7° (*c* 0.92, $CHCl_3$). HPLC-MS: Gradient from 15 to 95% ACN/H_2O (0.05% TFA) in 15 min, t_R: 5.17, (m/z: 521.69 $M+H^+$). HRMS (ESI pos) m/z Calculated $C_{25}H_{30}ClFN_4O_5$ 520.18888, found520.18863 (-0.47 ppm). ^{1}**H RMN** (400 MHz, $CDCl_3$): δ 8.07 (br t, 1H, CONH), 7.99 (s, 1H, 1-H), 7.21 (dd, *J* = 8.7, 6.0 Hz, 1H, Ph 6-H), 7.07 (dd, *J* = 8.6, 2.6 Hz, 1H, Ph 3-H), 6.93 (ddd, *J* = 8.7, 7.9, 2.6 Hz, 1H, Ph 5-H), 5.16 (s, 1H, 4-H), 4.59 (dd, *J* = 14.5, 6.4 Hz, 1H, 2-CH_2), 4.46 (dd, *J* = 14.5, 6.7 Hz, 1H, 2-CH_2), 3.56 (s, 3H, OCH_3), 3.49 (dd, *J* = 7.6, 5.2 Hz, 1H, Pen 2-H), 2.43 – 2.26 (m, 2H, Pen 4-H), 2.12 (ddd, *J* = 14.2, 7.4, 5.2 Hz, 1H, Pen 3-H), 2.05 (s, 3H, 6-CH_3), 1.93 (br s,

2, Pen 2-NH_2), 1.85 (td, *J* = 14.2, 7.6 Hz, 1H, Pen 3-H), 1.45 (s, 9H, ^{t}Bu CH_3). **^{13}C RMN** (101 MHz, $CDCl_3$): δ 177.6, 172.6, 167.2 (CO), 161.4 (d, *J* = 249.4 Hz, Ph 4-C), 146.6, 145.7 (2-C, 6-C), 139.3 (d, *J* = 3.5 Hz, Ph 1-C), 133.1 (d, *J* = 10.4 Hz, Ph 2-C), 131.6 (d, *J* = 8.8 Hz, Ph 6-C), 119.1 (5-CN), 116.9 (d, *J* = 24.4 Hz, Ph 3-C), 114.9 (d, *J* = 21.2 Hz, Ph 5-C), 101.6 (3-C), 85.7 (5-C), 81.1 (^{t}Bu C), 54.5 (Pen 2-C), 51.6 (3-$COOCH_3$), 39.0 (2-CH_2), 37.6 (4-C), 31.9 (Pen 4-C), 30.2 (Pen 3-C), 28.2 (^{t}Bu CH_3), 18.4 (6-CH_3).

S2.3. Acylation of primary amides

Following the methods included in the manuscript section 4.2.2.

Methyl (4S)-2-{[2-(benzyloxycarbonyl)amino-5-tert-butoxi-5-oxopentanamide]methyl}-4-(2-chloro-4-fluoro)phenyl-5-cyano-6-methyl-1,4-dihydropyridine-3-carboxylate (22ab). From **18** and Z-Glu(O^{t}Bu)OH. Method B: Yield 57%, it is obtained as diasteroisomeric mixture that could not be isolated. Eluent: Hexane:EtOAc (1:1). HPLC-MS: Gradient from 15 to 95% ACN/H_2O (0.05% TFA) in 10 min, t_R = 9.16, (m/z: 655.57 M+H$^+$). HRMS (ESI pos) m/z Calculated $C_{33}H_{36}ClFN_4O_7$ 654.22566, found 654.22655 (1.36 ppm). **^{1}H NMR** (400 MHz, $CDCl_3$): δ 7.67 (t, *J* = 11.9 Hz, 2H, Pen CONH), 7.32 (m, 14H, 1-H, Cbz Ph), 7.31 (m, 1H, H-1), 7.24 - 7.17 (m, 2H, Ph 6-H), 7.09 - 7.03 (m, 2H, Ph 3-H), 6.99 – 6.90 (m, 2H, Ph 5-H), 5.99 - 5.86 (m, 2H, Cbz-NH), 5.23 - 5.00 (m, 8H, 4-H, Cbz CH_2, NHCO), 4.60 – 4.33 (m, 4H, 2-CH_2), 4.18 (m, 2H, Pen 2-H), 3.53 (m, 6H, OCH_3), 2.46 - 1.83 (m, 10H, 2-CH_3, Pen 3-H, 4-H), 1.51 – 1.32 (m, 18H, ^{t}Bu CH_3). **^{13}C NMR** (101 MHz, $CDCl_3$): δ 174.5, 173.7, 167.3(CO), 161.3 (d, *J* = 249.2 Hz, Ph 4-C), 156.7, 156.6 (Cbz CO), 146.1, 145.73, 145.69 (2-C, 6-C), 139.4 (d, *J* = 3.1 Hz, Ph 1-C), 136.0 (Cbz Ph C), 132.9 (d, *J* = 10.1 Hz, Ph 2-C), 131.6 (d, *J* = 8.8 Hz, Ph 6-C), 128.72, 128.70, 128.6, 128.5, 128.4, 128.3, 128.2, 128.1 (Cbz Ph CH), 119.0 (CN), 116.8 (d, *J* = 24.3 Hz, Ph 3-C), 115.0 (d, *J* = 20.9 Hz, Ph 5-C), 101.2, 101.1 (3-C), 86.1 (5-C), 81.6, 81.2 (^{t}Bu C), 67.4, 67.2 (Cbz CH_2), 55.3, 55.2 (Pen 2-C), 51.61, 51.60 (OCH_3), 39.9, 39.8 (2-CH_2), 37.3 (4-C), 31.8 (Pen 4-C), 28.1 (^{t}Bu CH_3), 27.5, 27.3 (Pen 3-C) 18.24, 18.21 (6-CH_3).

S2.4. Hydrolysis of *tert*-butyl esters

Methyl 4-(2-chloro-4-fluoro)phenyl-5-cyano-2-[(malonamide)methyl]-6-methyl-1,4-dihydropyridine-3-carboxylate (21). The corresponding tert-butyl esters DHP **20** (50 mg, 0.1 mmol) was dissolved in a mixture 1:1 of CH_2Cl_2/TFA (1 mL). After 2 h of stirring at room temperature, the solvent was evaporated to dryness, with coevaporations with CH_2Cl_2. Then, the resulting residue was dissolved in a ACN/H_2O and it was lyophilized. In this conditions **21** was obtained with quantitative yield as a syrup. HPLC-MS: Gradient from 15 to 95% ACN/H_2O (0.05% TFA) in 10 min, t_R = 4.83, (m/z: 422.56 M+H$^+$). HRMS (ESI pos) m/z Calculated $C_{19}H_{17}ClFN_3O_5$ 421.08408, found 421.08286 (-2.88 ppm). **^{1}H NMR** (400 MHz, DMSO-*d*$_6$): δ 8.98 (s, 1H, 1-H), 8.40 (br t, 1H, NHCO), 7.37 (dd, *J* = 8.9, 2.6 Hz, 1H, Ph 3-H), 7.33 (dd, *J* = 8.6, 6.2 Hz, 1H, Ph 6-H), 7.20 (ddd, *J* = 8.6, 8.4, 2.6 Hz, 1H, Ph 5-H), 5.06 (s, 1H, 4-H), 4.39 (dd, *J*= 15.5, 5.5, 1H, 2-CH_2), 4.34 (dd, *J*= 15.5, 5.7, 1H, 2-CH_2), 3.47 (s, 3H, 3-$COOCH_3$), 3.27 (d, *J* = 3.3 Hz, 2H, Pr CH_2), 3.17 (s, 1H, COOH) 2.02 (s, 3H, 6-CH_3). **^{13}C NMR** (101 MHz, DMSO-*d*$_6$): δ 170.3, 166.8, 166.2 (CO), 160.5 (d, *J* = 247.0 Hz, Ph 4-C), 146.8, 146.6 (2-C, 6-C), 140.2 (d, *J* = 3.5 Hz, Ph 1-C), 131.8 (d, *J* = 9.4

Hz, Ph 6-C), 131.4 (d, *J* = 10.2 Hz, Ph 2-C), 119.1 (5-CN), 116.0 (d, *J* = 24.4 Hz, Ph 3-C), 115.4 (d, *J* = 20.9 Hz, Ph 5-C), 99.3 5-C, 83.9 (3-C), 51.2 (OCH_3), 42.5 (CH_2), 39.1 (2-CH_2), 36.6 (4-C), 17.5 (6-CH_3).

S3. Biological evaluation

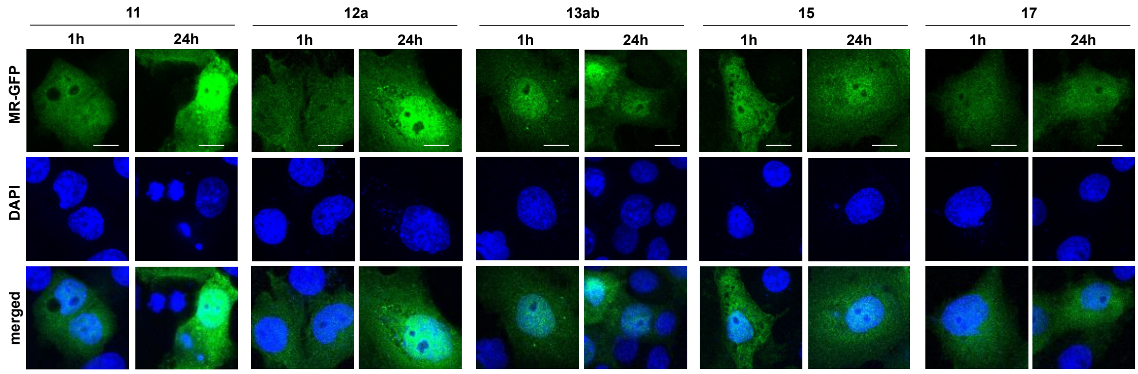

Supplementary Figure S1. Effect of the indicated 1,4-DHP derivatives on MR subcellular localization. See Fig.6 legend for experimental details. White bars, 10 μm.

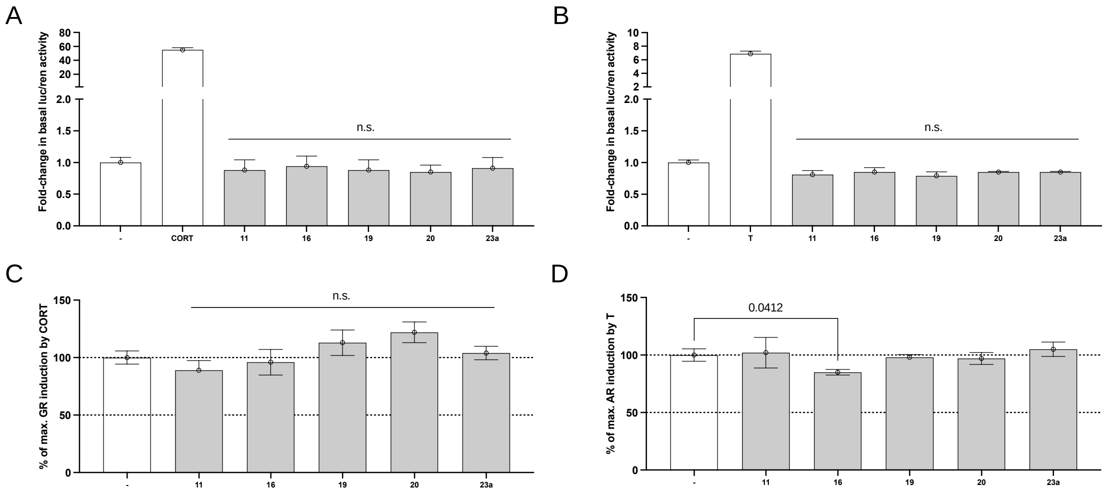

Supplementary Figure S2. Functional effects of 1,4-DHP derivatives on GR and AR transactivation properties. Bars represent the average value obtained from three technical replicates in one assay ± SE. Statistical analysis was performed with one-way ANOVA followed by Dunnett multiple comparison test where the average of each group was compared to the average of control (unstimulated) condition; n.s., not significant. A. Effects of 5 μM ligand treatment on basal GR activity. Induction of GR activity by 500 nM cortisol (CORT) is included to facilitate comparison. Averages were normalized to basal luciferase activity in the absence of any ligand. B. Effects of 5 μM ligand treatment on basal AR activity. Induction of AR activity by 10 nM testosterone (T) is included to facilitate comparison. Averages were normalized to basal luciferase activity in the absence of any ligand. C. Effect of 1,4-DHP derivatives (5 μM) on GR stimulation by 500 nM cortisol. Averages were normalized to maximal luciferase activity induced by 16h of treatment with the agonist. D. Effect of 1,4-DHP derivatives (5 μM) on AR

stimulation by 10 nM testosterone. Averages were normalized to maximal luciferase activity induced by 16h of treatment with the agonist.

S4. Molecular dynamics studies

Representative distances and angles along the 500 ns simulation for derivatives **11**, **19** and **20** are included in Figures S3-S6.

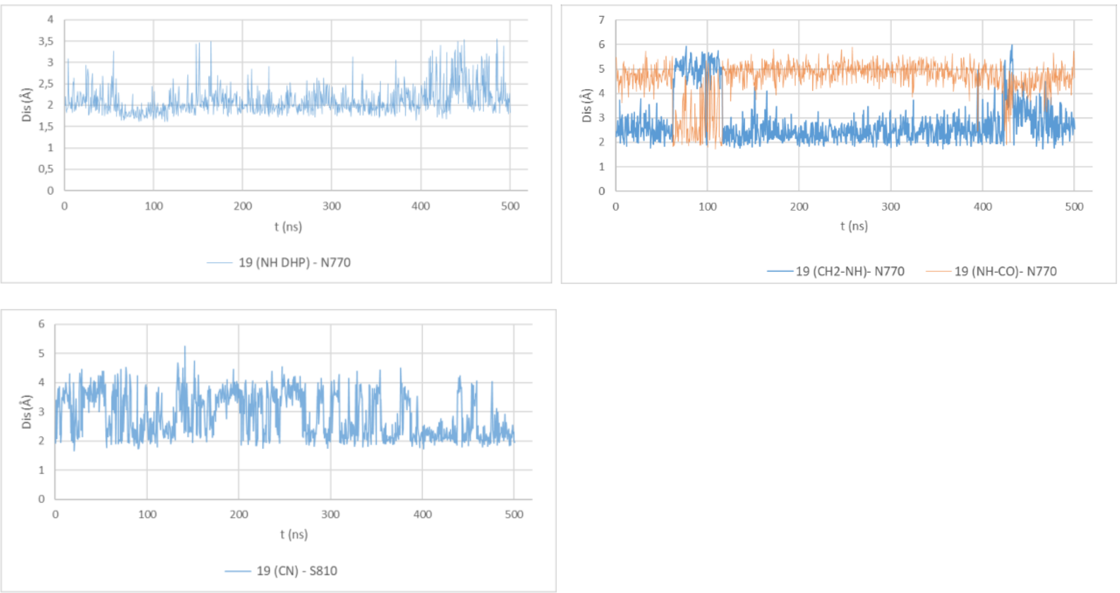

Supplementary Figure S3. Representative distances along the 500 ns MD between selected 1,4-DHP **19** and MR atoms.

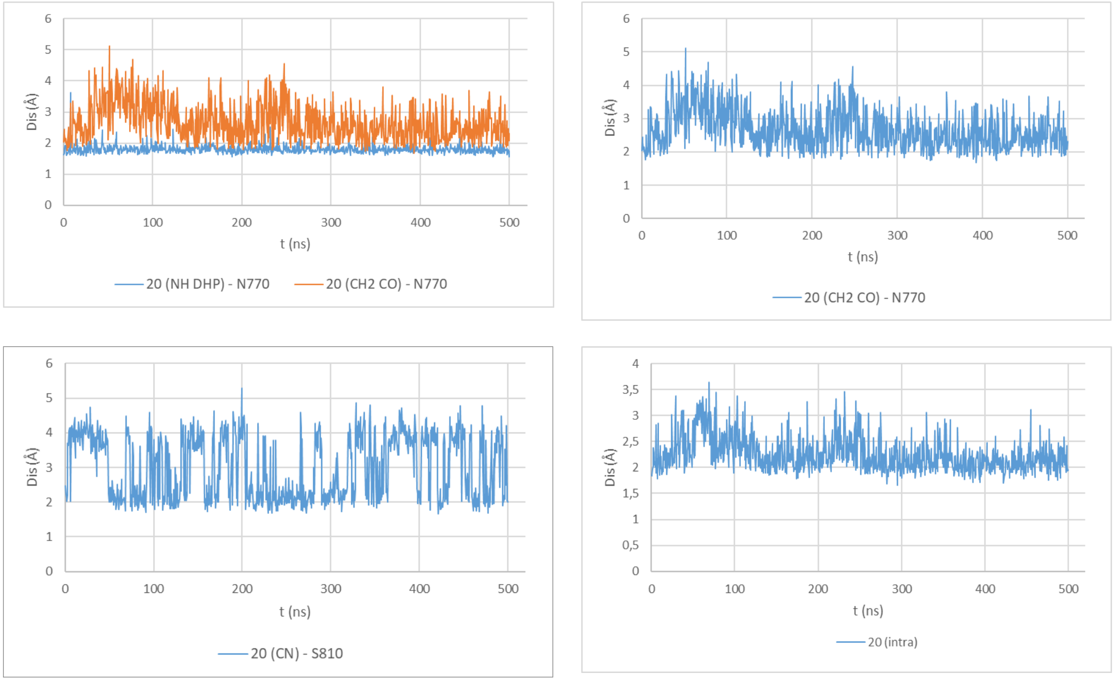

Supplementary Figure S4. Representative distances along the 500 ns MD between selected 1,4-DHP **20** and MR atoms.

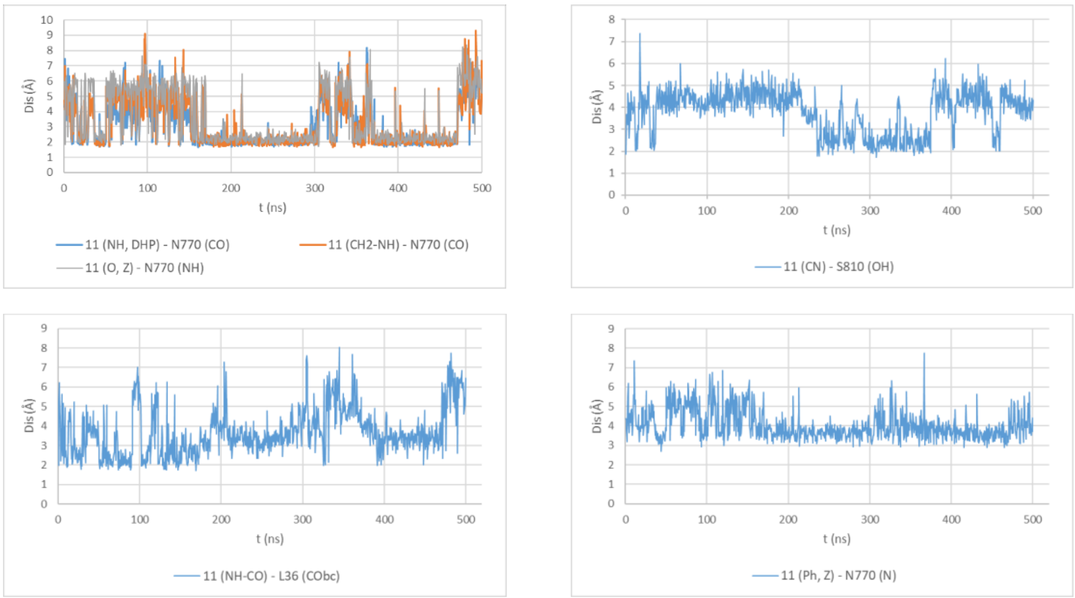

Supplementary Figure S5. Representative distances along the 500 ns MD between selected 1,4-DHP **11** and MR atoms. bc indicates backbone, otherwise it refers to atoms in amino acid side chains.

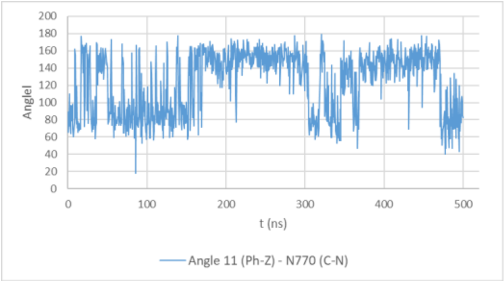

Supplementary Figure S6. Representative angle along the 500 ns MD between the centroid of the benziloxicarbonyl phenyl group of 1,4-DHP **11**, and the N-C atoms of N770 sidechain.

References

1. Cory, R. T.; Charles, K. Z., Chain extension of amino acid skeletons: preparation of ketomethylene isosteres. *Tetrahedron* **2003,** 59, (9), 1521-1527.
2. Hashiguchi, S.; Kawada, A.; Natsugari, H., Baker's Yeast Reduction of N-Protected Methyl 4-Amino-3-oxobutanoates and 3-oxopentanoates. *Synthesis* **1992,** 1992, (04), 403-408.
3. Katritzky, A. R.; Wang, Z.; Hall, C. D., Synthesis of Achiral and Chiral N-Protected γ-Amino-β-Ketones and β-Ketoesters. *ARKIVOC* **2007,** 2008, (10), 26-36.
4. Liang, B.; Richard, D. J.; Portonovo, P. S.; Joullie, M. M., Total syntheses and biological investigations of tamandarins A and B and tamandarin A analogs. *J Am Chem Soc* **2001,** 123, (19), 4469-74.
5. Perez-Faginas, P.; Aranda, M. T.; Garcia-Lopez, M. T.; Infantes, L.; Fernandez-Carvajal, A.; Gonzalez-Ros, J. M.; Ferrer-Montiel, A.; Gonzalez-Muniz, R., Highly functionalized 1,2-diamino compounds through reductive amination of amino acid-derived beta-keto esters. *PLoS One* **2013,** 8, (1), e53231.
6. Yao, Y.; Fan, W.; Li, W.; Ma, X.; Zhu, L.; Xie, X.; Zhang, Z., Synthesis of (S)-7-amino-5-azaspiro[2.4]heptane via highly enantioselective hydrogenation of protected ethyl 1-(2-aminoaceto)cyclopropanecarboxylates. *J Org Chem* **2011,** 76, (8), 2807-13.
